# Erythema Dyschromicum Perstans After Adalimumab Treatment

**DOI:** 10.7759/cureus.32264

**Published:** 2022-12-06

**Authors:** Ghazal G Alsisi, Mohammed H Alsisi, Alauldin K Alhowaish, Wardah S Alshammari

**Affiliations:** 1 Medicine and Surgery, Taibah University, Medina, SAU; 2 Dermatology, King Fahad Hospital, Medina, SAU; 3 Dermatology, King Fahad Hospital, Madinah, SAU; 4 Dermatology, King Fahad Hospital, Madina, SAU

**Keywords:** tumor necrosis factor (tnf), psoriasis vulgaris, erythema dyschromicum perstans, cutaneous hyperpigmentation, adalimumab

## Abstract

Tumor necrosis factor (TNF) is a cytokine that regulates immunity by binding to the cytokine receptor (TNFR), which has a role in treating inflammatory, neoplastic, and autoimmune diseases. Medications, including etanercept, infliximab, and adalimumab, are examples of TNF-alpha blockers. Adalimumab is a fully human immunoglobulin monoclonal antibody approved for use in the treatment of psoriasis, psoriatic arthritis, rheumatoid arthritis, inflammatory bowel disease, and hidradenitis suppurativa according to the American College of Rheumatology. However, there are few reports of cases where its administration was associated with skin reactions. In the present paper, we report a case of a psoriatic male patient who developed a cutaneous reaction of the face following treatment with adalimumab.

## Introduction

Psoriasis vulgaris is a chronic, relapsing, immune-mediated skin condition; this relapsing course necessitates lifelong therapy [1]. Treatment choices for psoriasis depend on several factors, including the severity of the disease, comorbid conditions, and accessibility to medical care [2]. The discovery of the immunopathogenesis of psoriasis has led to the advancement of therapies targeting the immune system that are responsible for inducing and maintaining the pathological and clinical abnormalities seen in psoriatic plaques. Biologic medications are a subset of a class of medications called disease-modifying antirheumatic drugs (DMARDs) that are derived from recombinant DNA technology, hybridomas, blood, and blood components [3]. In psoriasis, biologics usually target three main pathways: the inhibitors of interleukin-12 (IL-12) and interleukin-23 (IL-23), interleukin-17 (IL-17), and the inhibitors of tumor necrosis factor-alpha (TNF-alpha) [4]. TNF-alpha blockers are known to cause a variety of cutaneous adverse reactions, including eczematous dermatitis, alopecia areata, melanocytic lesions, and cutaneous symptoms of systemic lupus erythematosus [5]. TNF-alpha blockers include etanercept, infliximab, and adalimumab. In the present article, we report the case of a psoriatic male patient who developed a cutaneous reaction with hyperpigmentation of the face following treatment with adalimumab.

## Case presentation

A 36-year-old man with an 18-year history of chronic plaque psoriasis developed a rare cutaneous reaction with hyperpigmentation of the face following treatment with adalimumab (Humira). Before treatment, he had had moderate psoriasis of the skin with a Psoriasis Area and Severity Index (PASI) of 21.6. For several years, he had been treated with topical therapies, including corticosteroids, vitamin-D derivatives, and keratolytics. However, no improvement was noted, necessitating the use of an alternative therapy. He did not have any chronic diseases or significant family history. He did not take other medication, including herbal or over-the-counter medication. He smoked e-cigarettes, and he did not consume alcohol.

**Figure 1:**
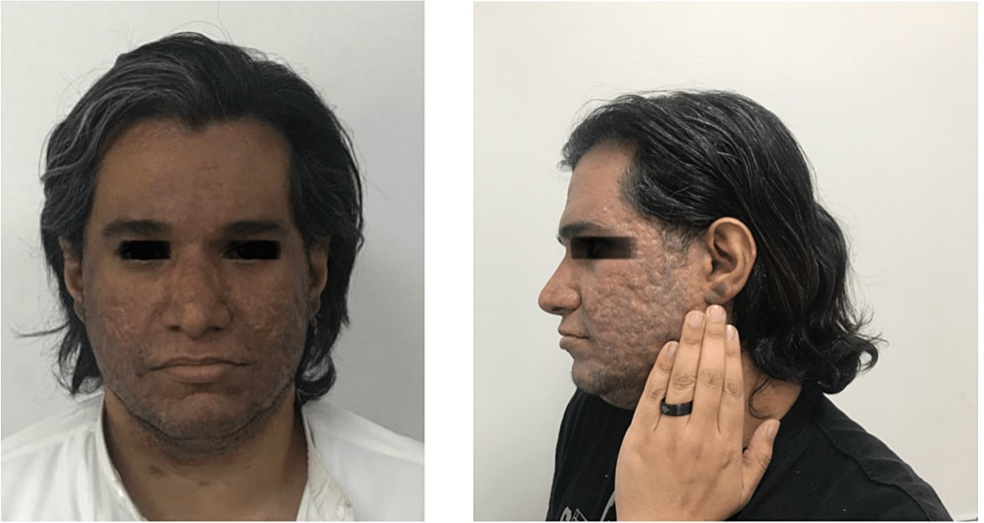
Hyperpigmentation of the face and auricular area. The skin on the hands is unchanged. Published with patient consent.

He began taking adalimumab 40 mg subcutaneously every second week. He improved after approximately three months, with a PASI of 5.7. One year after the initiation of adalimumab treatment, the patient noticed non-pruritic hyperpigmentation of the face and auricular area without any new activity during the day (Figure 1). No history of prolonged sun exposure exists. The patient did not experience any other symptoms. Physical examination revealed asymptomatic brown patches on the face and auricular area. There is acne scarring before adalimumab treatment. There were no other skin, nail, or oral lesions.

**Figure 2:**
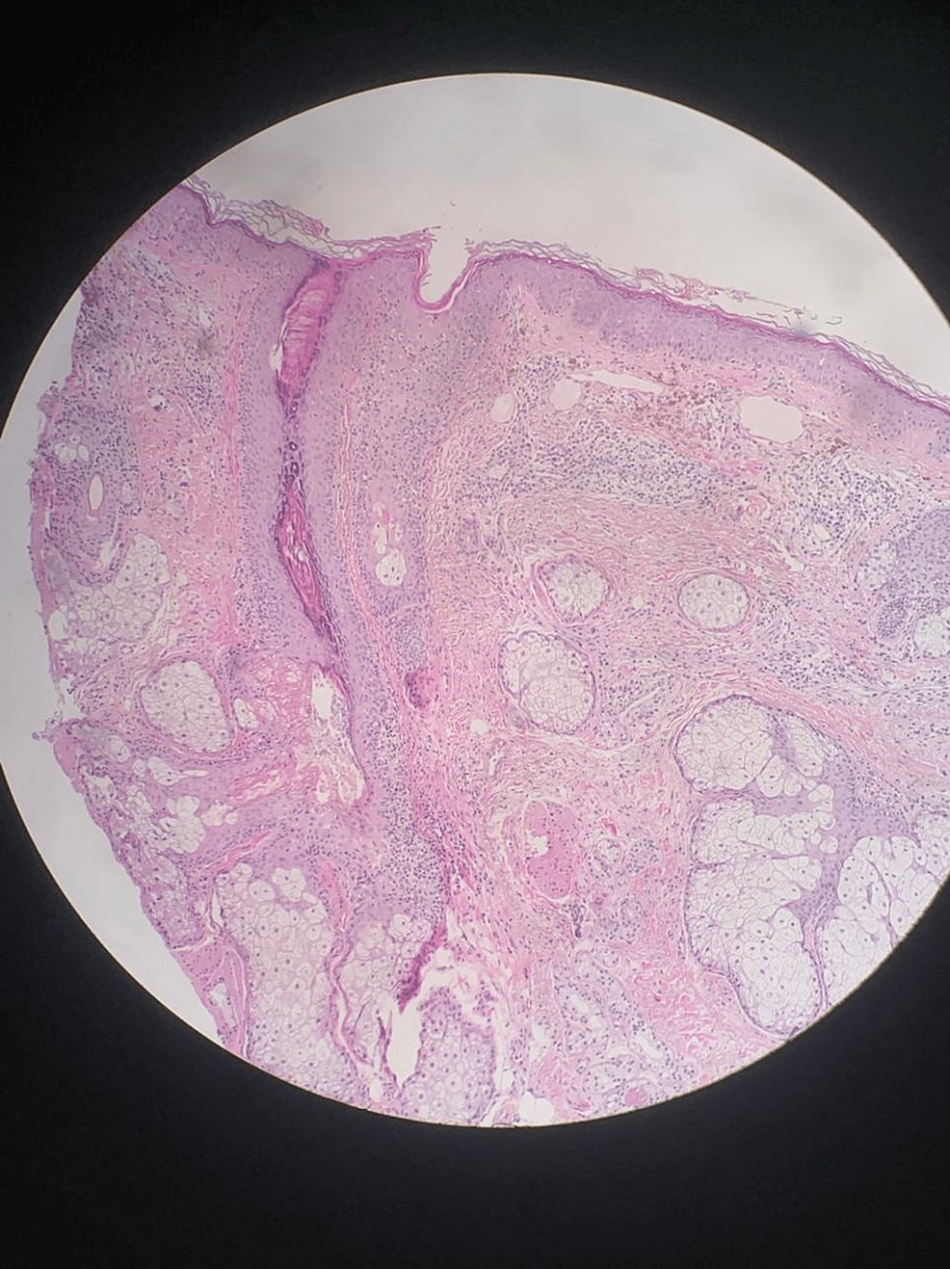
Histopathological feature: mild orthokeratosis, focal vacuolar alteration of basilar keratinocytes, and a superficial dermal infiltrate of lymphocytes, histocytes, plasma cells, and a few pigmented histocytes.

Complete blood count (CBC), liver enzymes, renal function, lipid profile, C-reactive protein (CRP), erythrocyte sedimentation rate (ESR), and hemoglobin A1C (HBA1c) were all within normal range. Antinuclear antibodies (ANA), DNA-antibodies (Anti-dsDNA), rheumatoid factor (RF), anti-SSA (RO), and anti-SSB (LA) were negative. Histopathological assessment of a 3 mm skin biopsy from the right cheek showed mild orthokeratosis, focal vacuolar alteration of basilar keratinocytes, and a superficial dermal infiltrate of lymphocytes, histocytes, plasma cells, and a few pigmented histocytes, indicating mild interface dermatitis with pigment incontinence (Figure 2). Thus, the diagnosis of erythema dyschromicum perstans was established. 

**Figure 3:**
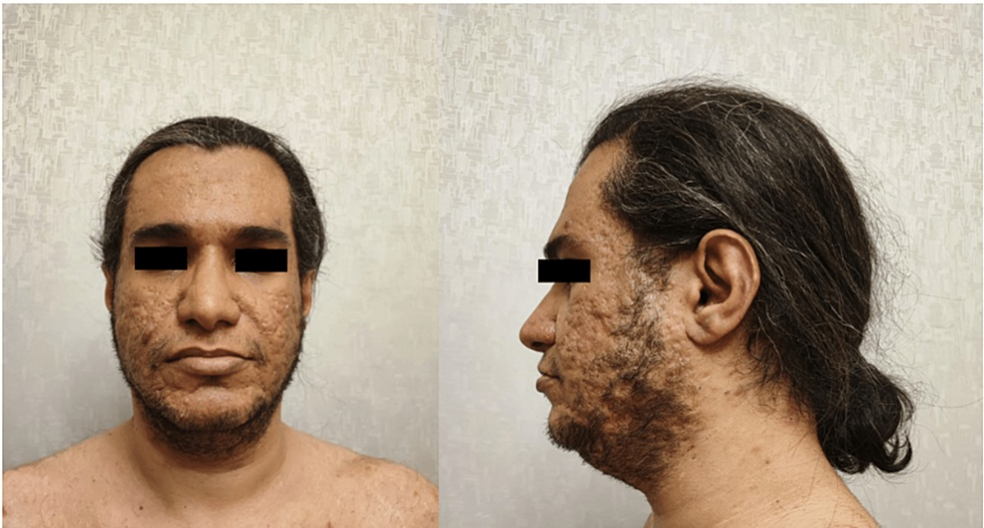
Clinical appearance of the hyperpigmentation improvement in the face and auricular area six months after the discontinuation of adalimumab. Published with patient consent.

The hyperpigmentation persists despite using hydroquinone, sunblock, and pimecrolimus. He was switched to ustekinumab in December 2021. Six months after the discontinuation of adalimumab, the hyperpigmentation dramatically improved without any residual lesions (Figure 3).

## Discussion

Erythema dyschromicum perstans is also known as ashy dermatosis [6]. Erythema dyschromicum perstans lesions appear as asymptomatic, symmetrical, grayish-ashy patches with peripheral erythematous margin, 1-2 mm in size. The trunk, proximal arms, and legs are the most often affected areas, sparing the scalp, mucous membranes, and the palms and soles. The pathogenesis of erythema dyschromicum perstans is still unknown. Several predisposing factors have been cited, including hypothyroidism, HLA-DR4 allele carriers [7], infections such as whipworm infestation [8], human immunodeficiency virus [9], chronic hepatitis C infection (HCV) [10], toxic substance exposure such as ingestion of ammonium nitrate [11], contrast media [12], and medication such as ethambutol [13], chlorothalonil [14], and omeprazole [15].

TNF is a cytokine that regulates immunity and is necessary for immunological functions. TNF-alpha blockers have inhibitory effects on TNF-alpha by binding to the cytokine receptor (TNFR), which has a role in treating inflammatory, neoplastic, and autoimmune diseases [16]. Adalimumab is a fully human immunoglobulin (IgG1) monoclonal antibody. It is administered by subcutaneous injection, approved for use in the treatment of psoriasis and psoriatic arthritis, rheumatoid arthritis, inflammatory bowel disease, and hidradenitis suppurativa [17]. According to the American College of Rheumatology, adalimumab is better than other TNF-blocker treatments in terms of the 70% improvement criteria and PASI [18]. Adalimumab has been shown to be superior to other TNF-alpha-blocking therapies in clinical studies [18]. The most common skin reaction related to TNF alpha blockers is adalimumab-related injection site reaction which occurs in 20.9%. TNF-alpha blockers have been linked to a variety of lichenoid eruptions, including cutaneous and oral lichen planus, maculopapular lichen planopilaris, and psoriasis-like lichen planus in the majority of cases [19]; urticaria, psoriasis flare-ups, lupus erythematosus, pustular dermatoses, and leukocytoclastic vasculitis can also occur; however, the majority of skin reactions improved once the medication was discontinued [20].

TNF-alpha blocker-induced cutaneous reactions with various mechanisms were reported in a few case studies. The hyperpigmentation, in this case, improved after six months of adalimumab discontinuation. Based on correlation and chronological connection, adalimumab may contribute to the pathogenesis of hyperpigmentation. Adalimumab is a type of systemic immunosuppressant that has been linked to an increase in melanocyte activity [19]. In addition, laboratory evaluations revealed that no significant findings were found. This patient was not predisposed physiologically to hyperpigmentation development. Sun-induced hyperpigmentation is less likely to be the etiology in this case as it is restricted to a small part of the face and auricular region without affecting the hands, arms, and neck, which are the areas most exposed to the sunlight.

## Conclusions

In conclusion, the purpose of the current case is to illustrate skin reactions associated with adalimumab therapy and to promote additional structural research in order to understand the mechanism of these reactions and correctly assess the relevance of this reaction. Additionally, clinicians should be aware of the uncommon adverse effects of adalimumab to monitor the patient's skin reaction and prescribe protective measures.
